# Designing Child Nutrition Interventions to Engage Fathers: Qualitative Analysis of Interviews and Co-Design Workshops

**DOI:** 10.2196/57849

**Published:** 2024-05-30

**Authors:** Jeffrey Tsz Hei So, Smita Nambiar, Rebecca Byrne, Danielle Gallegos, Kimberley A Baxter

**Affiliations:** 1 Centre for Childhood Nutrition Research Faculty of Health Queensland University of Technology Brisbane Australia; 2 School of Exercise and Nutrition Sciences Faculty of Health Queensland University of Technology Brisbane Australia

**Keywords:** co-design, fathers, child nutrition, child feeding, intervention design, digital delivery, parenting, participatory, videoconference, communication technology

## Abstract

**Background:**

Fathers play a pivotal role in parenting and child feeding, but they remain underrepresented in intervention studies, especially those focused on disadvantaged populations. A better understanding of fathers’ experiences and needs regarding support access and child nutrition information in the context of disadvantage can inform future interventions engaging fathers.

**Objective:**

This study aims to explore fathers’ experiences; perceived enablers; and barriers to accessing support and information related to parenting, child feeding, and nutrition and to co-design principles for tailoring child nutrition interventions to engage fathers.

**Methods:**

Australian fathers of children aged 6 months to 5 years with lived experience of disadvantage participated in semistructured interviews and co-design workshops, primarily conducted via videoconference. Creative analogies were used to guide the ideation process in the workshops.

**Results:**

A total of 25 interviews and 3 workshops (n=10 participants) were conducted, with data analyzed using reflexive thematic analysis and the Capability, Opportunity, and Motivation–Behavior model. The interview data illuminated factors influencing fathers’ initiation in seeking support for parenting, child feeding, and nutrition, including their experiences. It highlighted fathers’ diverse information needs and the importance of an inclusive environment and encouragement. Enablers and barriers in accessing support related to parenting and child nutrition were identified at the individual (eg, personal goals and resource constraints), interpersonal (family support and false beliefs about men’s caregiving role), organizational (inadequate fathering support), and systemic levels (father-inclusive practice and policy). Digital data collection methods enabled Australia-wide participation, overcoming work and capacity barriers. Videoconferencing technology was effectively used to engage fathers creatively. Key principles for engaging fathers were co-designed from the workshop data. Interventions and resources need to be father specific, child centered, and culturally appropriate; promote empowerment and collaboration; and provide actionable and accessible strategies on the *what* and *how* of child feeding. Fathers preferred multiformat implementation, which harnesses technology-based design (eg, websites and mobile apps) and gamification. It should be tailored to the child’s age and targeted at fathers using comprehensive promotion strategies.

**Conclusions:**

Fathers faced barriers to accessing support and information related to parenting and feeding that may not adequately address their needs. Future interventions could integrate the co-designed principles to engage fathers effectively. These findings have implications for health service delivery and policy development, promoting father-inclusive practice.

## Introduction

### Background

Nurturing care is a central tenet for fostering optimal growth and development in children. This supports children in attaining good health through adequate nutrition, feeling safe and secure, and receiving responsive caregiving and learning opportunities [1]. Child feeding that focuses on the reciprocal positive relationship between the caregiver and the child is an opportunity that integrates all components of nurturing care. Efforts to promote early childhood development recommend incorporating responsive caregiving as part of interventions aimed at optimizing the nutrition of young children [2].

Fathers play a pivotal role in nurturing care, including feeding, and influencing children’s eating [1,3]. Although mothers often take on the primary responsibility for feeding children, fathers increasingly participate in various aspects of child feeding, from selecting and preparing foods to sharing family meals [4-6]. Despite an emerging trend of involving fathers in parenting and child health research, there is limited evidence documenting fathers’ experiences and needs regarding nutrition and feeding, and they remain underrepresented in intervention studies [7,8]. In a 2017 systematic review of randomized controlled trials (RCTs) targeting child obesity prevention and treatment (n=213), only 10% of participants were fathers, and just 2 studies reported targeted attempts to recruit fathers [9].

A barrier to the inclusion of fathers is their reported reluctance to participate in research even when invited, as they may doubt they have contributions to make to studies on children’s eating [10]. On the basis of their own research experience, Moura and Philippe [10] found that fathers with a lower socioeconomic background expressed discomfort with being involved in research, perceiving researchers as *too knowledgeable* to provide additional insight into their work. In another survey study (n=303), >80% of fathers perceived their underrepresentation in child health research as stemming from not being invited to participate in these studies [11]. Fathers living with disadvantage can be considered as *hard to reach* because of structural barriers, including work commitments, limited financial resources, low literacy, or reduced capacity to travel [12]. Consequently, researchers have tended to focus on populations that are easier to reach, typically more advantaged mothers. Targeted strategies are necessary to make participation more accessible and engaging for fathers, particularly those facing disadvantage. Remote and digital data collection methods emerge as promising tools to engage with disadvantaged populations, breaking structural barriers to inclusive participation [13]. The nature of remote technologies, in which participants operate within their settings and control their devices and degree of involvement, also creates a safe environment and balances power dynamics between researchers and participants.

Understanding how to tailor research and service design, including intervention objectives and content, delivery mode, and location, is crucial for the effective engagement of fathers. Moura and Philippe [10] identified practical facilitators to enhance the inclusion of fathers in child nutrition research, including explicitly recruiting *fathers* rather than *parents*, offering web-based participation options, and using interactive methods. However, these suggestions have emerged from reflection and review rather than directly drawing on the lived experiences of fatherhood. To develop tailored interventions that will optimize child nutrition and maximize success in reaching fathers from diverse backgrounds, participatory research, in which fathers are considered experts in their lived experience, may produce more concrete and realistic solutions.

### Objectives

Understanding fathers’ experiences and needs regarding child nutrition interventions in the context of disadvantage can inform future intervention development. This research aimed to understand the following: (1) fathers’ experiences; perceived enablers and barriers in accessing support and information related to parenting, child feeding, and nutrition and (2) how child feeding and nutrition interventions can be effectively tailored to engage fathers through co-design.

## Methods

### Ethical Considerations

Both studies were approved by the Queensland University of Technology (QUT) Human Research Ethics Committee (2022-5253-7746 and 2023-6687-16117).

### Context

The data presented in this paper come from the research project Dads at Mealtimes (DAM), which consisted of 3 phases: a web-based survey (phase 1), interviews (phase 2), and co-design workshops (phase 3). The overarching research explored the feeding roles and practices of Australian fathers in the context of disadvantage. The survey and interviews were conducted in 2022, informing the subsequent co-design workshops in 2023. All studies primarily used digital tools because of the COVID-19 pandemic, during which digital data collection became more common. This paper focuses on findings from the interview and workshop phases.

The interviews aimed to explore three aspects: (1) paternal roles and contributions in child feeding, (2) enablers and barriers to responsive feeding practices, and (3) experiences and perceived enablers and barriers in accessing support and information related to parenting, child feeding, and nutrition. Objectives 1 and 2 have been reported elsewhere [14]. This paper focuses on data from the interviews, which contribute to objective 3.

**Figure 1:**
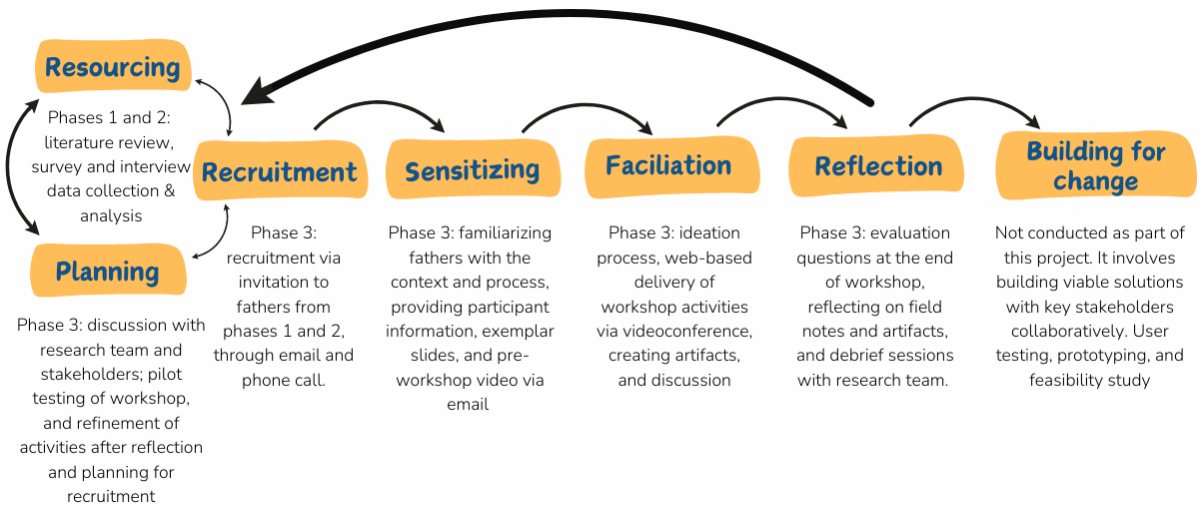
Overview of the co-design framework and mapping to the study phases and activities of the Dads at Mealtimes project.

The co-design workshops aimed to gain insight into how child feeding and nutrition interventions can be effectively tailored to engage fathers. The workshop uses co-design as a participatory approach, methodology, and method that includes end users in the intervention’s conception, development, or evaluation [15]. Underpinned by the principles of equity and partnerships [16], co-design seeks to build capacity, harness creativity, and deepen collaboration between professionals and people experiencing or impacted by the issues [17-19]. Thus, co-design has the potential to create socially and contextually appropriate solutions aligned with the circumstances and contexts of end users [20]. The DAM project aligns with the Kennedy et al [21], extended model of the co-design framework originally developed by Trischler et al [22]. The framework is inherently iterative, with an evolving and flexible design process of resourcing, planning, and recruitment, which is reflective of different study phases and multiple workshops. An overview of the 7 steps and their alignment with the DAM study phases is depicted in Figure 1 [21,22].

Phases 1 and 2 of the DAM study served as the first step (resourcing) in Figure 1 to gain an understanding of the issue. Of the 264 fathers who completed the survey, two-thirds reported having prepared meals (67%) and assisting their child with eating (69%) at least once a day. More than three-quarters (77%) of participants were food insecure, and 55% reported having unmanaged stress [23]. From the interviews (objectives 1 and 2), personal, interpersonal, and systemic enablers (eg, food skills, adequate resources, and support) and barriers (eg, low self-efficacy in feeding, financial and mental strain, food insecurity, and gendered stereotypes) were identified as influencing paternal feeding experiences [14]. The insights from phases 1 and 2 informed the workshop’s scope, aim, and activities for engaging fathers in child nutrition interventions. These findings underscored the need for intervention design that harnessed fathers’ lived experiences to provide tailored strategies.

### Theoretical Framework

The conceptual framework for the broader study incorporates the Capability, Opportunity, and Motivation–Behavior (COM-B) model of behavior change [24]. This model conceptualizes *capability*, *opportunity*, and *motivation* as the main components for facilitating behavior change. Adopting this behavior change perspective provides a basis for highlighting key attributes in engaging fathers in child nutrition. Consequently, it integrates findings from phases 2 and 3 to inform intervention design.

### Positionality

The researchers’ positionality is of pivotal importance as coconstructors of meaning in both studies. The first author (JTHS), a male PhD student, conducted recruitment and performed data collection and analyses for both the interview and workshop studies. Not a father himself but involved in caring for young children within an extended family household, he has a background in nutrition and dietetics and completed training in qualitative research. The coauthors (KAB, SN, RB, and DG) are academic dietitian-nutritionists specializing in child nutrition and have experience in qualitative research and co-design. They are mothers of children spanning a broad age range. None of the authors had any prior relationship with the participants.

### Interviews

#### Recruitment

Recruitment procedures for participating in DAM have been detailed previously [14]. Briefly, participants self-identified as fathers or male caregivers with a child aged 6 months to 5 years living with disadvantage. The following question served as an indicator of socioeconomic disadvantage: do you sometimes struggle to pay the bills? The screening question was informed through consultation with parents with the aim of using language that sensitively recruited individuals who were struggling financially and, therefore, at risk of food insecurity [25]. Interview participants were predominantly recruited from a pool of participants who completed the survey (phase 1) and expressed interest in future research. In addition, participants were recruited via promotional flyers distributed to stakeholders, such as family and child services. Consent and demographic information were obtained through a brief web-based questionnaire. All web-based data collection tools were developed using REDCap (Research Electronic Data Capture; Vanderbilt University) [26,27], which was hosted by QUT.

#### Data Collection

A semistructured interview guide was informed by a literature review and the COM-B model. Pilot testing of the interview questions was completed with 3 fathers to check for comprehension and flow. These questions explored paternal perceptions of their roles and feeding practices, experiences of food insecurity, and accessing support and child nutrition information [14]. Indicative questions relevant to this paper’s findings were as follows: (1) Can you tell me any advice you received from anyone or anywhere about child feeding and nutrition? (2) Do you think you received enough support and information about child nutrition? The decision on the sample size is guided by the concept of *information power*, which posits that the greater the relevant information the sample provides, the fewer participants are required [28]. Participants were compensated with an Aus $25 (US $16.6) e-gift card. All interviews were recorded and transcribed verbatim using an automated transcription service, Otter.ai [29]. Participants were invited to review the transcripts. Those who opted to receive the transcripts proposed no alterations. Debriefing sessions were conducted with the research team during data collection and analysis to foster theoretical and reflective thoughts.

#### Analysis

A detailed description of the data analysis is reported elsewhere [14]. The analysis used the 6-phase process of reflexive thematic analysis [30]. The first author conducted manual coding using inductive and deductive approaches underpinned by a symbolic interactionism lens [31]. Coauthors independently coded a subset of transcripts (n=3) and engaged in discussions to sense check ideas and refine themes. The codes and themes derived from the interview data allowed the researchers to identify enablers and barriers to support access among fathers across individual, interpersonal, and systematic levels. This analysis process was guided by the COM-B model, which was integrated with the workshop findings.

### Co-Design Workshops

This section of the paper outlines the recruiting, planning, sensitizing, facilitation, and reflecting steps for the co-design workshops. As part of sensitizing, participants who completed the interview (phase 2) received a summary of findings before phase 3 data collection.

#### Recruitment

**Figure 2:**
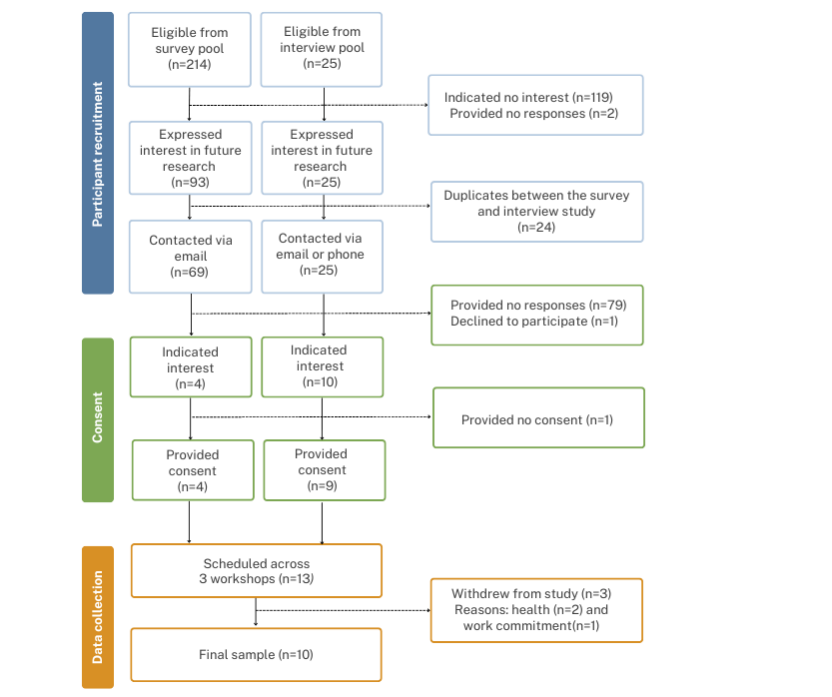
Workshop participant flow diagram, including reasons for exclusion.

Workshop participants were recruited from a pool of fathers who completed either phase 1, phase 2, or both and expressed interest in further research. Purposeful sampling was adopted to optimize participation from those who completed both phases. Given the sequential design, rescreening for eligibility was not completed. Fathers were invited to participate via email or telephone and were informed that workshops involved several creative web-based activities to design solutions to engage fathers in child feeding and nutrition. Consent and participants’ availability were collected via a web-based questionnaire using REDCap [26,27], which was hosted by QUT. The participant flow diagram is presented in Figure 2.

#### Data Collection

The first step in developing the workshop guide was a literature review on fathers’ participation in parenting and child nutrition research [10,11,32] and co-design methodologies [19,33]. Discussions were then held among research team members with expertise in child nutrition, socioeconomic disadvantage, and co-design. This was followed by a consultation with a design practitioner-academic with expertise in co-design. After these discussions, a decision was made to conduct workshops of shorter duration and with more streamlined ideation activities focusing on paternal engagement. Drawing on the first author’s experience with other co-design studies and sense checking the ideas with other fathers (not part of the final sample), creative analogies that resonate with men were deemed appropriate. The workshop using *superhero* analogies was pilot-tested with 3 fathers (not included in the final sample). These fathers considered the activities and analogies acceptable and enjoyable in eliciting creative ideas. They provided feedback on simplifying the language in the materials (eg, workshop guide, visual slides, and videos) and suggested probes and examples to enhance the concepts and understandability. The workshop overview is provided in Textbox 1.

The preliminary sample size for the workshops was 8 to 10, guided by *information power* [28]. The plan included conducting 2 to 3 workshops, each lasting 70 minutes, with 3 to 4 fathers in each session to facilitate small group discussions. To enable participation across Australia and provide flexibility, workshops were conducted via videoconference using the Zoom platform (Zoom Video Communications) [34]. This reduced barriers to participation and met fathers’ preference for web-based activities [10,35]. Features of the platform that were used included screen share, audiovisual recording, and live chat, and it was compatible with mobile or computer use. The *superhero*-themed workshop was complemented by a short preworkshop animated video sent to participants 3 days before the workshop. This video aimed to familiarize fathers with the context, objective, activities, and exemplar personas. These personas were presented as comic characters, incorporating key attributes and findings from phases 1 and 2 (refer to Multimedia Appendix 1 for exemplar personas and related presentation slides).

**Figure 3:**
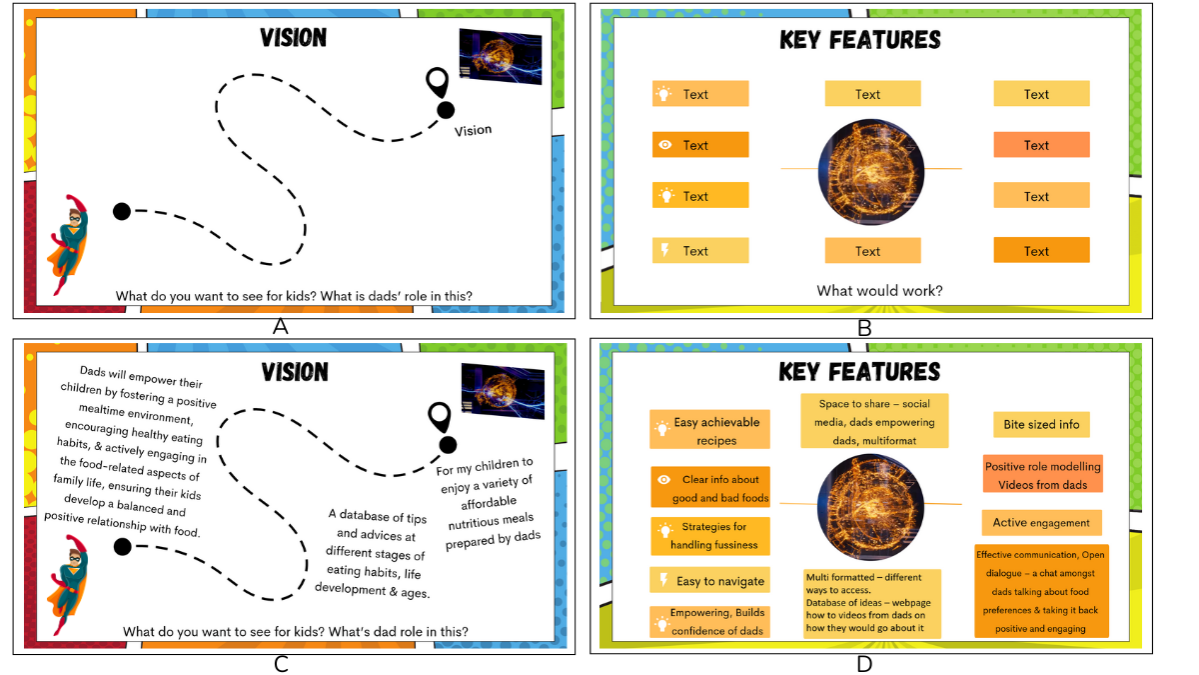
Exemplar slides: (A) template slide for identifying the vision, (B) template slide for identifying key features, (C) completed slide of participants’ vision (workshop 1), and (D) completed slide of identified key features (workshop 1).

Each workshop was facilitated by the first author (JTHS), who was one of the coauthors acting as a scribe (DG, KAB, and SN). A presentation slide guided the ideation activities throughout the workshop. Activity 1 involved creating their persons guided by exemplar personas (refer to image C in Multimedia Appendix 1 for 1 father’s drawing of their persona). Fathers were asked to ideate their vision and key features of designed solutions in activity 2. Figures 3A and 3B demonstrate the exemplar slides used in this activity. The screen-sharing feature allowed facilitators to share the slides to guide the ideation process and enter responses (provided verbally or via chat) onto these slides in real time for participants to view, validate, and elaborate (refer to Figures 3C and 3D for generated presentation slides). Participants were encouraged to use the live chat function to contribute to the activities or ask questions. At the end of each workshop, the cofacilitator gave a summary of the discussion, providing an opportunity for participants to validate and add further comments. Evaluation questions were posed (ie, What do you like most and least? What would you change?), serving as a short reflection to conclude the workshop. This allowed researchers to refine procedures between the workshops. After the first workshop, this process resulted in a modification to send participants sample slides and questions ahead of time. Participants received an Aus $30 (US $19.9) e-gift card for their involvement.

All workshops were video-recorded and transcribed verbatim using Otter.ai [29]. The first author (JS) also completed field notes on each workshop. Artifacts, including drawings, transcripts of chat conversations, and presentation slides (ie, visions and key features created on the slide with fathers), were collated. The research team held debriefing sessions throughout the data collection and analytic process.

**Workshop overview and activities.**

**Overview**

- Study objective: to co-design tailored child nutrition intervention design principles for engaging fathers
- Approach: creative analogies using the *superhero* theme
- Workshop name: Superdads: The New Age of Nourishing Kids
- Workshop activities and indicative questions
  Activity 1: making superheroes (15 minutes)
    Description: fathers created their personas, guided by exemplars.
    Tools: screen sharing of visual slides, drawing, and group sharing.
    Indicative question: Tell us about yourself (family role, education, cultural background, and life experience), alias (superhero name), your kids, a tip of advice (superpower), challenges (evil nemesis), and information sources.
  Activity 2: feeding into (fictional artificial intelligence name in the comic) (45 minutes)
    Description: Participants ideated their vision and brainstormed the key features of the designed solutions; ideas were refined with probes (considerations and challenges informed by phases 1 and 2).
    Tools: live chat function, group discussion, and presentation slide with real-time responses (ie, facilitator entering words on slides).
- Indicative questions
  What do you want to achieve for dads and kids regarding child nutrition and eating? (collective vision).
  What are your ideas for possible solutions for dads, what would work? What are your top 3 design features if we create something for dads about child nutrition? Why are they important?
  What format could these solutions take on the basis of these features?
- Probes
  Consider space—where is the best place to reach dads?
  Consider time—when is the best time to target dads?
  Consider what empowers dads, their strengths, and challenges. Think about the personas we created.
  Consider the target group (father only, family focused, and children). Who do you trust to give credible information (male, peer, partner, general practitioner, and health professionals)?

#### Analysis

Underpinned by the constructivist paradigm that views reality as socially constructed, workshop data (video and chat transcripts, presentation slides, field notes, and artifacts) were analyzed using the same approach as the interviews with inductive coding. Specifically, the enablers and barriers constructed from the interview findings (objective 3) were interpreted together with the workshop data using the COM-B model. In this interpretive process, coauthors were involved in sense checking ideas, generating, naming, and defining the key themes (design principles).

## Results

### Demographics

A total of 25 fathers participated in semistructured interviews conducted from April to September 2022 (n=24, 96% completed the interviews digitally). Three co-design workshops were held with a sample of 10 fathers (3 or 4 fathers in each workshop) between October and November 2023. The demographics of participants are presented in Table 1. In total, 60% (6/10) of workshop participants completed both phases 1 and 2, and 40% (4/10) completed the survey (phase 1) only.

**Table 1:** Characteristics of the fathers participating in the interview and workshop studies.

Characteristics			Interviews (n=25)		Workshops (n=10)
Father’s age (years), mean (SD)			35 (6)		35 (4)
Biological father to the index child, n (%)			25 (100)		10 (100)
**Days living with the child per fortnight, n (%)**					
	Full time (14 days)	19 (76)		8 (80)	
	Part time (7 days)	3 (12)		1 (10)	
	Less than part time (2-4 days)	3 (12)		1 (10)	
**Marital status, n (%)**					
	Married or in a de facto relationship	18 (72)		9 (90)	
	Separated or divorced	6 (24)		1 (10)	
	Widowed	1 (4)		0 (0)	
**Cultural and ethnic group, n (%)**					
	Australian	18 (72)		8 (80)	
	New Zealander	1 (4)		1 (10)	
	Aboriginal or Torres Strait Islander	1 (4)		0 (0)	
	Other^a^	5 (20)		1 (10)	
**Education level, n (%)**					
	University	14 (56)		6 (60)	
	Certificate or diploma	5 (20)		3 (30)	
	Year 12	5 (20)		1 (10)	
	Year 7-9	1 (4)		0 (0)	
**Employment or study status, n (%)**					
	Employed^b^	20 (80)		9 (90)	
	Parental duties	3 (12)		0 (0)	
	Study or apprentice	2 (8)		1 (10)	
**Household composition, n (%)**					
	Single child	9 (36)		5 (50)	
	2 children	10 (40)		2 (20)	
	4-7 children	6 (24)		3 (30)	
**Number of adults^c^, n (%)**					
	1	4 (16)		0 (0)	
	2	20 (80)		9 (90)	
	3	1 (4)		1 (10)	
**Number of children (aged 0-5 years)^c^, n (%)**					
	0	0 (0)		1 (10)	
	1	13 (52)		7 (70)	
	2	10 (40)		2 (20)	
	3-4	2 (8)		0 (0)	
**Number of children (aged 6-17 years)^c^, n (%)**					
	0	18 (72)		6 (60)	
	1	3 (12)		1 (10)	
	2	1 (4)		1 (10)	
	3-6	3 (12)		2 (20)	

^a^Cambodian, Chilean, Congolese, Indian, and Indonesian for the interview sample; Cambodian for the workshop sample.

^b^For interviews, of the 25 participants, 14 (56%) were full-time, 1 (4%) was part-time, and 5 (20%) were casual employment. For workshops, employment type was not collected.

^c^The household size is reported for the father’s household.

### Interviews

#### Overview

The interview data illuminated fathers’ experiences in accessing support and information related to parenting, child feeding, and nutrition. These experiences are reflected by the following themes: (1) factors influencing the initiation of support related to parenting and child feeding, (2) experiences when seeking support, (3) diverse information needs, and (4) inclusive environment and encouragement. ID numbers are assigned to participants’ quotes to preserve anonymity.

#### Factors Influencing the Initiation of Support Related to Parenting and Child Feeding

Fathers reported multifaceted factors that influenced their likelihood to seek and access support *in relation to parenting and child feeding*. These included resource constraints, such as time, location, high mental load, and traditional masculine values, when seeking help might be regarded as a weakness. When asked about their experiences obtaining support around feeding children, 1 father stated the following:

As a dad, you don’t want to ask for help, it’s not a manly thing to do.

ID64

How fathers perceived their fatherhood roles and responsibilities regarding caregiving or feeding guided whether and how they sought information. Some fathers believed caregiving to be instinctual (a *gut instinct*) and perceived nutrition as *common sense*. Thus, they are driven by responding to the emotions and behaviors of their child rather than seeking external advice. Others only sought information when they were looking for specific nutrition knowledge. For example, a father indicated the following:

When we face questions we didn’t know...we tried to solve it early, went to GP, went to nutritionist at the time.

ID122

Awareness of available support and gendered expectations, in which fathers perceive and abdicate the responsibility of seeking health and nutrition information to mothers, also influenced whether they sought external support:

My wife does like all the research...she drives what we’re doing—Okay, we’re going to move on to more solid foods or feed her this...she’s part of a lot of mother’s groups as well, it’s a lot of information from there and I just don’t have the time so there’s no point in doubling up.

ID61

#### Experiences When Seeking Support

When fathers sought child health information and feeding support, experiences were mixed. Although some fathers reported good support from health care workers, others recounted negative experiences in which they received minimal help. One father shared his experience after the birth of his baby:

No help for dads...I said—Could you show me how to wash her and all that, my wife couldn’t at the time and [the medical staff] said you have to wait until your wife is ready...she got a lot of help but nothing to teach the men how to feed a kid or change a nappy… at the moment I need the help, so teach me.

ID8

The stigma of fathers being perceived as uninvolved or unimportant was also raised:

There’s stigma about dads not being involved as much as they should be. But when you go into an appointment with the mum...you don’t exist. It’s like mum made the baby and you’re just brushed to the side.

ID118

In some instances, fathers reported feeling treated as auxiliary parents, in which child health information and systems are geared toward mothers who are designated as primary caregivers:

There’s information that when you go through the system, the support and consultations. [But] the system is obviously more focused on the mother, so if the mother is not in a state to absorb that information, there’s not as much information provided to the father.

ID74

Other fathers shared concerns about being judged and harbored distrust toward health professionals when seeking child health information. Information was regarded as *generic* or *overwhelming*. In 1 father’s words, he felt coerced and judged by health institutions:

You feel a lot of pressure from government agencies that if [the child is] not eating this, you’re not doing the right thing.

ID95

Highlighting the insufficient father-specific support in caregiving, feeding, and mental health, fathers expressed the importance of services to be sensitive to diverse family structures and care arrangements. One father indicated the following:

[I wish I have] more information about feeding, like how often do they feed, because my partner got that information, I got information of looking after [my] partner.

ID213

Such experiences could hinder fathers’ self-esteem, creating barriers to seeking further support and developing their capabilities in child feeding:

No mental health side for the dads. When things got tough, I had to take all the responsibilities on...There’s no one to talk to for my side of things.

ID8

#### Diverse Information Needs

Most fathers sought information on child nutrition and feeding from a range of sources, including family, health care providers, mass media (internet, television, and web-based videos) and social media, printed materials (leaflets and books), and community groups. The topics covered a broad spectrum, encompassing breastfeeding and bottle feeding, complimentary food, recipes, child appetite cues and eating behaviors, food safety, and allergies. Fathers often trusted their partners, deeming them as more organized and well informed. Family members, including grandparents and peers, acted as *sounding boards*, providing validation for ideas. Fathers also took measures to assess the reputability of the sources, such as using government websites. In addition, fathers value the expertise of health care workers who have a shared understanding of being a dad:

There was a doctor that was a dad, he gave us the rundown...you know, don’t worry, it’s just a dad thing. It was nice talking to a dad who is also a doctor.

ID 8

#### Inclusive Environment and Encouragement

To address fathers’ unique needs and promote paternal involvement in child health, an inclusive environment where fathers feel welcomed is crucial. One father stated the following:

A workshop with the kid [would] be beneficial as long as you don’t feel judged, [because] you are already feeling really vulnerable.

ID64

This vulnerability arises from experiencing financial and food insecurity while navigating fatherhood despite perceived gender stereotypes regarding parenting roles. Although some fathers discussed the need for self-determination in caregiving and feeding, others believed that child health initiatives and professionals should play a role in actively advocating for fathers’ involvement, building relationships, and dismantling traditional gender stereotypes:

Having an ad campaign [and] for the midwife or paediatrician or obstetrician [to] brought up at the start of the pregnancy that it is going to be helpful if dad comes. When they get letters to attend appointments, have it addressed to the mum and the dad or says dad is encouraged to attend.

ID118

There was also a call for mothers to encourage fathers to fulfill the caregiver role and participate in feeding. For example, a father said the following:

If you’re a mum, it’s okay to tell your partner, that dads are allowed to do things.

ID3

### Enablers and Barriers to Support Access

**Figure 4:**
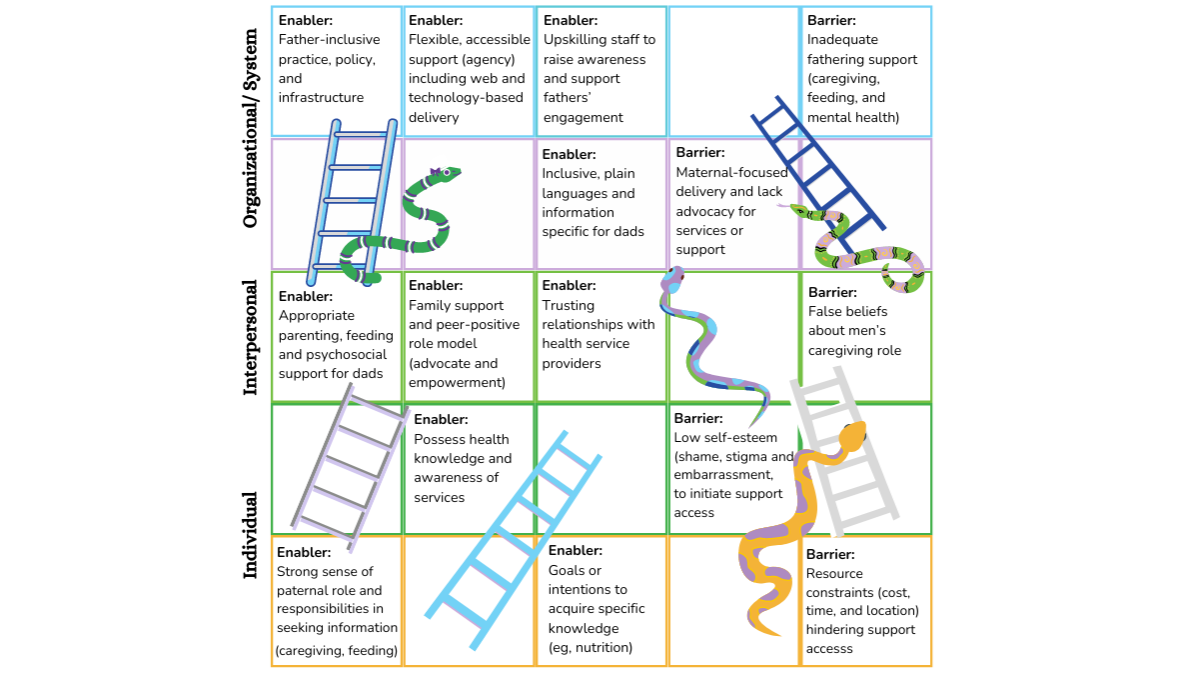
Enablers and barriers for accessing support: presented as snakes and ladders.

Figure 4 presents the interrelated enablers and barriers influencing fathers’ access to support across individual, interpersonal, organizational, and system levels. The interviews first provided insights into fathers’ enablers and barriers. The workshops allowed for more in-depth exploration, focusing on how to overcome barriers and leverage enablers through co-designed solutions. These factors were embedded into the workshop videos and activities, including the exemplar personas and presentation slides, to facilitate the ideation process.

### Co-Design Workshops

#### Overview

**Figure 5:**
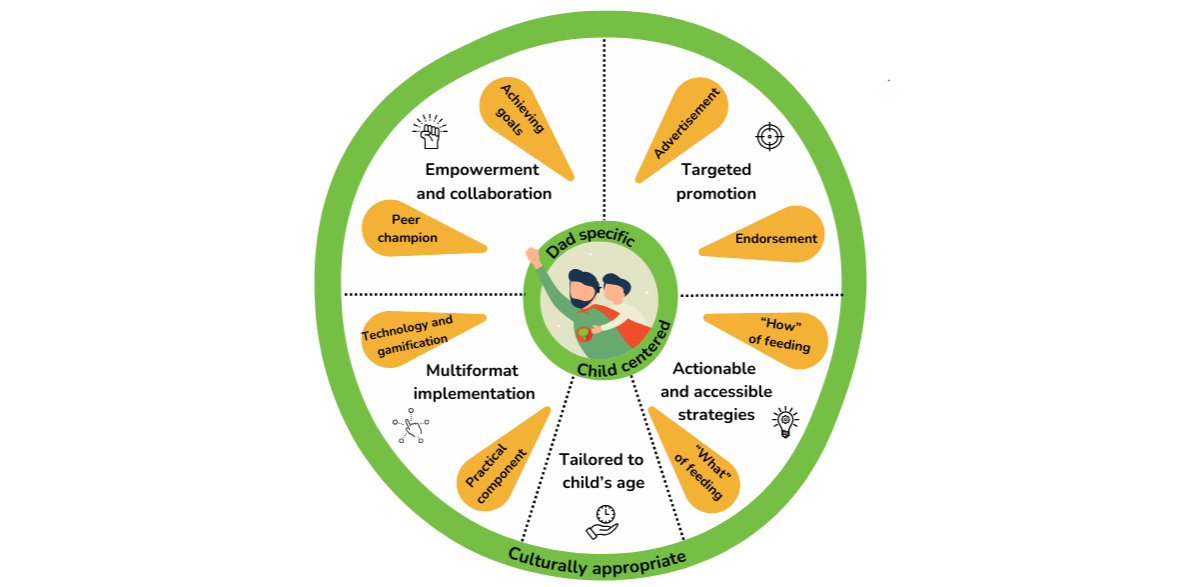
Design principles for engaging fathers in child nutrition interventions.

From the workshop data, 7 principles for engaging fathers in child nutrition interventions and resources were identified. These include (1) father specific and child centered, (2) empowerment and collaboration, (3) actionable and accessible strategies, (4) multiformat implementation, (5) culturally appropriate, (6) tailored to the child’s age, and (7) targeted promotion. Illustrative quotes of each principle, along with the superhero name (if provided) or pseudonym and workshop number, are presented in Multimedia Appendix 2. These design principles encompass various aspects of interventions, from recruitment to content development, and are visually presented in Figure 5.

#### Father-Specific and Child-Centered

Fathers identified the need for child-centered interventions specifically designed for fathers themselves, given that most current food and nutrition content predominantly target mothers. Proposed *hooks* for actively engaging fathers in nutrition interventions included strengthening family connections through food, promoting personal growth, and enhancing father-child relationships. This involves supporting fathers to create affordable and nutritious meals, model healthy eating habits, and foster children’s positive relationship with food. In 1 father’s words, it is important that “dads lead by example [for their child], showing enthusiasm for healthy eating and trying new foods” (W1, Captain Aus). Interventions should involve the child in providing bonding opportunities, such as through “engaging recipes [so] the child can partake in cooking” (W2, Dr Strange). Personalization can support active engagement, which includes providing personalized meal plans and recipes and feedback on performance through using technologies such as mobile apps. Others described web-based tailored support with a moderator who would be able to drive conversations and respond to inquiries within father group chats:

Whoever monitors the dad groups—got to keep it positive to try and engage everyone. A lot of people join up to them, but no one comments [or] wants to go first, so if you’re there positively backing up comment...The more people comment, you can get an active involved community...

W1, Hulk

#### Empowerment and Collaboration

Interventions should harness peer empowerment and collaboration to facilitate fathers’ engagement. Fathers discussed the collective goals of addressing “the stigma that dads are not as good as mums” and being “proud to be a good dad and doing the best for our kids” (W3, Captain Wellness). Creating an interactive fathers’ community to exchange ideas about child eating and demonstrate role modeling was thought to be a powerful avenue to build trust and confidence. Seeing other father ambassadors who “do not know how to cook” and realizing “their struggles are the same” was considered valuable:

It does [motivate you and empower fathers’ identity] if we can create something...I have a family, I’m a father, but I’m independently able to do it...I cook an amazing meal and everyone eats it, it’s like “damn right, I did that.” It’s good for the soul...

W1, Superdad

Fathers identified the opportunity for empowerment through self-monitoring and incentivizing behaviors, such as tracking progress and earning badges when mastering a food ingredient and technique or budgeting skills. The ability to act as a peer champion, coaching novice fathers or competing with others, was considered effective in fostering collaboration:

I’m always driven to be better than I was yesterday...I see cooking as my own development...In a digital environment, I would look for ways that show progress...like last 12 months, I’ve made x amount of meals, and gone from beginner to more advanced or experience more recipes or more ingredients in a way that I can sort of compete with myself and my peers.

W2, Dr Strange

#### Actionable and Accessible Strategies

Fathers described the necessity for bite-sized and actionable strategies regarding the *how* and *what* of child feeding and eating. This includes guidance on teaching nutrition-related information to their child, improving basic food skills (eg, gardening and cooking), and addressing common concerns around infant feeding (eg, allergies and food safety) and fussy eating behaviors:

Showing dads how different foods can improve your kids in different ways, like your kids need to have this because it can help with bones or be more active like this. If they don’t have it, it may affect them like that.

W2, Panther

Fathers highlighted the importance of variety and simple recipes for an enjoyable meal preparation experience for the whole family. They discussed providing clear instructions and using language that resonates with fathers (eg, *recipes presented as a tech manual* and *building a meal*). Accessibility is crucial, considering factors such as time, location, cost, and literacy. Interventions that use technology and build fathers’ capacity might overcome these challenges:

With time poor, budget constraints and income pressures, [the app, website, or workshop] sort of met the ideas of gaining control...and your competence in making [the meal]. So you sort of alleviate those stresses...

W2, Flash

#### Multiformat Implementation

Fathers sought to move beyond traditional handouts to use technology-based delivery for more personalization. Suggestions included popular social media platforms to host group discussions and websites and mobile apps to provide child nutrition information. These elements should be complemented with behavioral prompts that provide simple educational messages or reminders to prompt action (eg, fridge magnets in prominent places or email reminders). In addition to information provision, practical components, such as video or in-person cooking workshops involving children, remain critical. Gamification could be integrated to enhance engagement, adding an interactive and playful dimension:

It would have to be multiformat. One format may not work. The more engagement you can get whether it is one central platform, then you run workshops off the side to get engagement. That’s [what] I would enjoy more.

W1, Superdad

I see an app supported by a website, [which] give the same info, but the app would gamify it...This makes it fun, something my son and I can work towards together. He understands games and that keeps him engaged...[like] list of foods to try, get rewards when we try them.

W3, Thor

#### Culturally Appropriate

Food embodies connections, experiences, and culture. Interventions should consider cultural diversity, family dynamics and values, the cost of food, and the skills of those doing the preparation, recognizing that the significance of food extends beyond nourishment to encompass cultural identity and shared experiences. For example, 1 father discussed the need for intervention design to be sensitive to cultural identity:

Different cultures, food and family dynamics...It’s how you brought up [and] your key beliefs. My wife eats certain foods that I don’t eat because of our backgrounds, so my children get to choose what they eat. So saying this is what we’re going to eat and this is the recipe, cook on this day wouldn’t work for everyone. I’d have to consider different backgrounds and cultures.

W3, Captain Wellness

#### Tailored to Child’s Age

Fathers discussed the provision of timely materials tailored to the development stages of children, considering the introduction of complementary foods and eating milestones (eg, supporting breastfeeding, age-appropriate recipes, transitioning food texture, and key nutrients). Some fathers suggested that it would be beneficial to provide resources on food allergies and food safety (eg, choking risks) as well as children’s capabilities to engage in different food tasks (eg, holding a knife) at different ages:

I guess my vision is a database...of tips, hints, and advice of different stages of eating and what to do or try. Obviously different stages of age and life development they go through different eating habits...

W1, Superdad

#### Targeted Promotion

Fathers described a comprehensive, father-targeted promotion strategy using various channels. Marketing was recommended in places traditionally directed at men (eg, hardware and sports venues), web-based platforms (eg, YouTube and Facebook) and community spaces (eg, childcare centers, sports venues, and grocery shops). They acknowledged the influential role of mothers in effectively reaching fathers. Framing of the intervention was key to appealing to fathers, specifically using the word *dads* and father images for promotion purposes. Fathers emphasized the value of endorsement from peers (*someone like me*) and experts (eg, celebrity chefs, health professionals, and academics), who are also fathers, to enhance relatability and trust:

I guess like [facilitator’s name], PhD, told me something, it’s good. I’d go along with it because you got the credential...I’m happy to follow the authorities and the research, peer reviewed studies...well, okay I will do that.

W1, Hulk

### Integration With the COM-B Model

**Figure 6:**
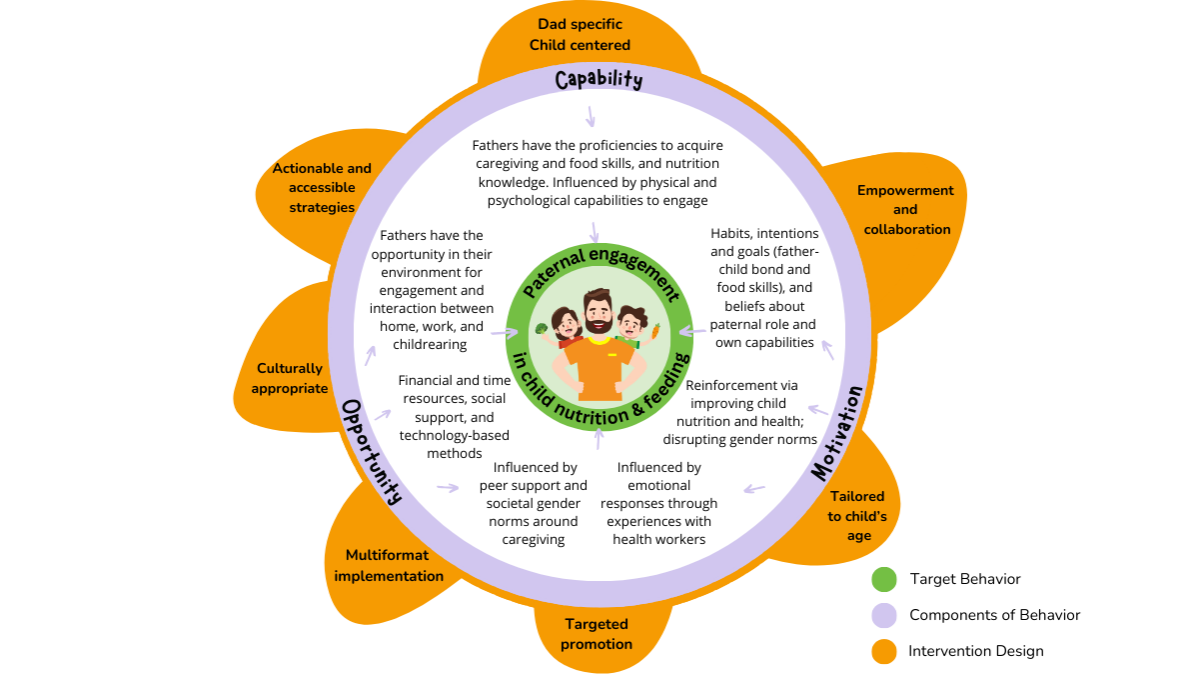
Intervention design: integrating findings with the Capability, Opportunity, and Motivation–Behavior model.

The COM-B model provided a theoretical lens to integrate findings to inform intervention design that facilitates fathers’ engagement in child nutrition. Figure 6 visually illustrates the underlying components of behavior change, encompassing capability (eg, physical and psychological capabilities to acquire food skills and nutrition knowledge), opportunity (eg, material and time resources and social support), and motivation (eg, intentions, goals, and reinforcement). The intertwined design principles can be leveraged to address each component: capacity (eg, through actionable and accessible strategies), opportunity (eg, via multiformat implementation and targeted promotion), and motivation (eg, by being father specific and fostering empowerment and collaboration).

## Discussion

### Principal Findings

This research offers valuable insight into fathers’ experiences when accessing support related to parenting and child feeding and nutrition information, recognizing the enablers and barriers at individual, interpersonal, organizational, and systemic levels. Fathers’ intrinsic motivation concerning fatherhood, trusting relationships, social support, and perceived usefulness of information are crucial for active paternal engagement in accessing support related to parenting and child feeding. Conversely, support access is hindered by adverse experiences with health services, fear of judgment, resource constraints (ie, time and location for face-to-face delivery), and gender expectations.

Key principles for child nutrition interventions were identified through co-design. The results indicated that interventions and resources need to be (1) father specific and child centered; (2) leverage empowerment and collaboration; (3) provide actionable and accessible strategies on the *what* and *how* of child feeding; (4) implemented in multiple formats, including technologies; (5) culturally appropriate for diverse practices and values; (6) tailored to child’s age and developmental needs; and (7) promoted directly to fathers to engage them effectively.

Drawing from the COM-B model, it is crucial to identify and address barriers when designing interventions to optimize parental feeding and nutrition in children. One significant barrier to fathers’ inclusion in public health nutrition initiatives is rooted in gender ideology. Traditional masculinities (reflecting *motivation* of behavior change), characterized by risk taking, invulnerability, plenitude, and autonomous decision-making [10,36], can impede fathers from seeking support in regard to child health and nutrition. These gendered traits may manifest in child feeding, in which the nurturing role or responsibility for child health is typically associated with maternal identity. In this study, some fathers considered healthy eating as *common sense*. Thus, these role expectations and perceptions may deter fathers from seeking information on their children’s nutrition.

Qualitative studies with fathers have shed light on the barriers to paternal involvement in child health and nutrition initiatives, such as the Special Supplemental Nutrition Program for Women, Infants, and Children in the United States [37]. The key challenges reported in this study include pride in masculinity, coercion, unacknowledged roles, and feelings of exclusion [37]. Although aiming to improve the health and nutrition of low-income women and children aged <5 years, only a few local offices strive to invite men to participate [38]. The Special Supplemental Nutrition Program for Women, Infants, and Children’s name, program structures, and staff-client interactions have been criticized for being unwelcoming and unresponsive to family dynamics and paternal roles [37]. Similarly, fathers in this study stressed the importance of encouragement, inclusiveness, and a father-specific environment (linking both *opportunity* and *motivation* of COM-B) as they navigate their fatherhood role. Thus, intervention designs should engage fathers as a distinct target demographic, using platforms and resources that resonate with men (eg, father imagery and wordings in the promotion and educational materials, technology-based activities, and suitable time outside of regular work hours when providing support).

Many fathers in this study actively sought information on child nutrition from various channels, including health care workers, family, peers, and the internet. Future interventions need to account for the diverse sources fathers seek information from by leveraging these platforms to reach fathers effectively. For instance, health care workers can raise awareness of intervention studies through their services, or mothers can act as agents to promote study information to fathers (*opportunity*). Accessing information through other fathers suggests the potential benefits of mobilizing peer empowerment as a resource to facilitate paternal engagement in child nutrition interventions (*motivation*). Participants in the workshops further expressed an interest in learning about nutrition (*capability*), particularly if their children are the focus. This child-centric approach is consistent with previous research [35,39]. For example, fathers preferred engaging in nutrition intervention with their children and the whole family rather than being targeted themselves [35]. Communicating a clear goal and benefits of fostering father-child bonding has been suggested to be valuable for parenting interventions targeting fathers [40]. This bonding opportunity can be applied to the feeding context, in which emphasizing positive father-child feeding interactions may enhance future intervention uptake.

This study highlights the underlying motivators for paternal engagement in child nutrition, including supporting fathers as role models for their children’s eating behaviors and building positive relationships with food (*capability*). Fathers clearly indicated a preference for participating in interventions *with* their children rather than *for* them, whether through in-person programs or technology-based platforms. The success of the *Health Dads, Healthy Kids* (HDHK) and *Healthy Youngsters, Healthy Dads* (HYHD) community-based RCTs in Australia serves as a renowned example internationally [41,42]. Both programs effectively engage fathers and children (aged 5-12 years in HDHK and aged 3-5 years in HYHD) in learning about healthy eating and physical activity [41,42]. Notably, these interventions yielded favorable effects on various outcomes, including improved fathers’ and children’s weight outcomes, physical activity levels, and dietary intakes [41,43-45]. A core feature of HDHK and HYHD is its family-based approach, which aims to improve men’s and children’s well-being simultaneously, focusing on fathers as positive role models and implementing effective parenting strategies. Incorporating practical and theoretical components, the programs offered activities and resources for fathers (eg, manual for dads) and children (eg, activity handbook) individually, as well as opportunities for joint participation (eg, rough and tumble play) [41,42]. HDHK and HYHD have been tailored to men using humor, language, and content that cater to their needs. In addition, they used behavior change techniques, such as monitoring, goal setting, and social support. However, these programs have been implemented with older children. Nutrition interventions involving young children may necessitate adaptations to match their developmental stages and warrant further investigation with fathers and other stakeholders.

In-person delivery, such as that offered by the HDHK or HYHD programs, could pose barriers for certain families, such as those with time and geographic constraints and work commitments. Indeed, fathers in this study emphasized there is no one-size-fits-all approach, advocating for multiformat implementation (*opportunity*). Interventions using technology, such as websites, web-based chat groups, social media, and mobile apps, were recognized as providing more pragmatic options while maintaining interactive elements. This is comparable to a survey study examining Australian fathers’ preferences for child nutrition interventions, in which web-based programs were deemed the most popular delivery mode, followed by written materials [35]. Similarly, the *Fathers Infant Feeding Initiative* reported paternal preferences for the internet, email, and video as the basis for delivering perinatal programs supporting breastfeeding [46]. Digital delivery offers a cost-effective and scalable format to provide family-based health programs while overcoming accessibility issues [47]. *Milk Man* is a father-focused app that exemplifies using mobile technology to provide social support and information about breastfeeding [48]. In their process evaluation involving 586 fathers, push SMS text messaging notifications and web-based conversation forums were found to be integral to the app’s success, prompting fathers to post comments and access articles and external links. One-third of users also indicated gamification as a key motivator for app use [49]. A previous review of RCTs using gamification found promising results in enhancing nutritional knowledge and dietary behaviors among children and adolescents [50]. Fathers in this workshop study similarly favored interactive games and visual content. Therefore, technology-based intervention designs can be expanded to target other areas of child nutrition. Additional research is crucial to examine the acceptability and feasibility of game-based interventions for fathers with young children.

In a systematic review of interventions designed to shift men’s attitudes and behaviors in relation to gendered stereotypes, interactive learning, co-design, and peer leadership emerged as cornerstones for maximizing impact [51]. In a recent practice article, Moura and Philippe [10] proposed recommendations for recruitment, focus, and methods to facilitate fathers’ engagement in child-feeding research. They advocated for culturally appropriate, child-focused interventions with a clear framing of the *father* and a focus on lived experiences, using participatory web-based activities and tailored and flexible materials. Several studies also recommended peer-based recruitment and messages (*someone like me*) based on fathers’ interests and characteristics (ie, small-time commitment and incentives) [10,11,40,52]. The co-designed principles derived from the workshops corroborate with these recommendations, incorporating insights from fathers experiencing disadvantage. An earlier study involving fathers with low-income status emphasized that nutrition education should focus on food as opposed to complex nutritional knowledge, as well as framing basic nutrition information positively [39]. Simple, actionable strategies that translate knowledge into practice were paramount for fathers in this study. Equally important is the provision of evidence-based information, demonstrated by their efforts to seek credible sources and experts’ advice. Collaborative input from fathers on content and messages, combined with professional expertise, can create solutions that prioritize relationships and maximize intervention impact.

These findings present new perspectives that diverge from existing research. For instance, Jansen et al [35] reported that fathers preferred a whole-of-family focus over a fathers-only program. This contrasted with this study, in which participants emphasized the need for a father-only community for sharing and peer encouragement. This was rationalized by their shared experience pertaining to fatherhood and their collective goal of addressing gender stereotypes. Although some fathers discussed sharing information with other family members (apps and websites), certain elements of the intervention (eg, chat groups) that remain father exclusive may be beneficial.

An SMS text message–based intervention has been shown to be acceptable in providing men breastfeeding support in Ethiopia [53] and in Australia, where programs such as *SMS4dads* offered perinatal support related to mental health and parenting [54]; this intervention mode was not raised by fathers in this study. However, digital modes of intervention emerged as a common thread in the discussions. Although SMS text messaging may not offer sufficient practicability for certain nutrition topics, such as food skills and recipes, communication through emails, text messages for promotion, and linked information is considered feasible. Intervention design would benefit from building on this co-design study to determine how individual components and formats can complement each other to elicit positive outcomes. This is of great importance for scaling up programs to be embedded into services and informing policies.

### Limitations

The interview and workshop studies have limitations that should be considered in interpreting the results. Fathers who took part in this research were less representative of the Australian male population in terms of education levels. National data indicate that approximately 38% of men aged 25 to 44 years held a university degree [55], a proportion lower than the 57% observed in this sample. Most participants were in relationships, resided with their child full time, and identified as Australian. The self-selection nature of the studies may inadvertently exclude fathers who are less involved, less interested in child nutrition, or more susceptible to entrenched disadvantage, such as single fathers, those with lower literacy, individuals who are unemployed and socially isolated, Indigenous Australians, or culturally and linguistically diverse communities. Future studies could use a more comprehensive sampling strategy to ensure diversity, including fathers with varying education levels, ethnicities, relationship statuses, family sizes, and relationships with the child (ie, biological vs social).

Furthermore, the workshop findings should be considered in light of the group setting and technology used. Individuals less inclined toward group discussion or lacking English language proficiency may not have participated. The study design may favor those who are familiar with using videoconference software. Consequently, these factors pose a potential limitation to the generalizability of the findings, particularly concerning preferences for technology-based and interactive interventions. Future research should strive to identify and recruit a more diverse range of male caregivers. Child nutrition interventions would gain from fathers’ perspectives from various family dynamics, such as same-sex fathers and nonbiological fathers and diverse cultural backgrounds, to co-design culturally appropriate engagement strategies.

### Implications for Research, Practice, and Policy

This research holds implications for practice, research, and policy. Amplifying fathers’ voices in child health research recognizes the developmental benefits of paternal involvement in nurturing care and optimal nutrition. Co-design, which harnesses the lived experience expertise of fathers facing disadvantage, strengthens their capacity to contribute to public health initiatives. Future interventions seeking to effectively engage fathers could incorporate the co-designed principles into their planning and delivery.

Although the primary aim of this research was to inform intervention design, the findings have the potential to be extrapolated for health service delivery and policy development. Existing evidence suggests that fathers encounter accessibility barriers when engaging with services. For example, a study on Australian fathers’ participation in antenatal care highlighted a gendered approach in providing parenting support, lack of knowledge and decision-making involvement, and paternal anxiety as notable barriers [56]. In addition, paternal depression symptoms were found to be linked to fathers’ perceived lack of support and poor father-child and coparent relationships [57]. The design principles identified in our workshops align well with best practice and father-inclusive guidelines, which advocate for a revaluation of how services are planned and delivered to be responsive to fathers’ needs and recognize their strengths [58,59]. The enablers and barriers identified provide valuable direction for parenting and child health services, policies, and infrastructure, especially in tailoring child nutrition information for fathers. These findings underscore the importance of a systemic approach to dismantle gender stereotypes, provide feeding and psychosocial support, and strengthen father-child relationships to achieve positive outcomes for children.

### Conclusions

Fathers encounter substantial barriers when accessing support and information related to parenting and child feeding, and existing resources are inadequate for their needs. To harness the lived experience of fathers, future interventions could incorporate the co-designed principles developed in this study to effectively engage fathers. These findings hold implications for health service delivery and policy development, advocating for practices that foster fathers’ engagement.

## Appendix

Multimedia Appendix 1 Exemplar slides on personas: (A) exemplar personas (comic characters) incorporating fathers’ characteristics from phases 1 and 2, (B) template slide for guiding making superhero activity, and (C) participants’ drawing (artifact) from the making superhero activity (workshop 3).

Multimedia Appendix 2 Design principles for child nutrition interventions with descriptions and illustrative quotes.

## Abbreviations

COM-B: Capability, Opportunity, and Motivation–Behavior
DAM: Dads at Mealtimes
HDHK: Health Dads, Healthy Kids
HYHD: Healthy Youngsters, Healthy Dads
QUT: Queensland University of Technology
RCT: randomized controlled trial
REDCap: Research Electronic Data Capture
