# Supplementary material for: Designing Child Nutrition Interventions to Engage Fathers: Qualitative Analysis of Interviews and Co-Design Workshops
Source: JMIR Pediatr Parent. 2024 May 30;7:e57849. doi: 10.2196/57849 (PMC11176881; doi:10.2196/57849)
Supplement: Multimedia Appendix 1 [file pediatrics_v7i1e57849_app1.docx]

Multimedia Appendix 1


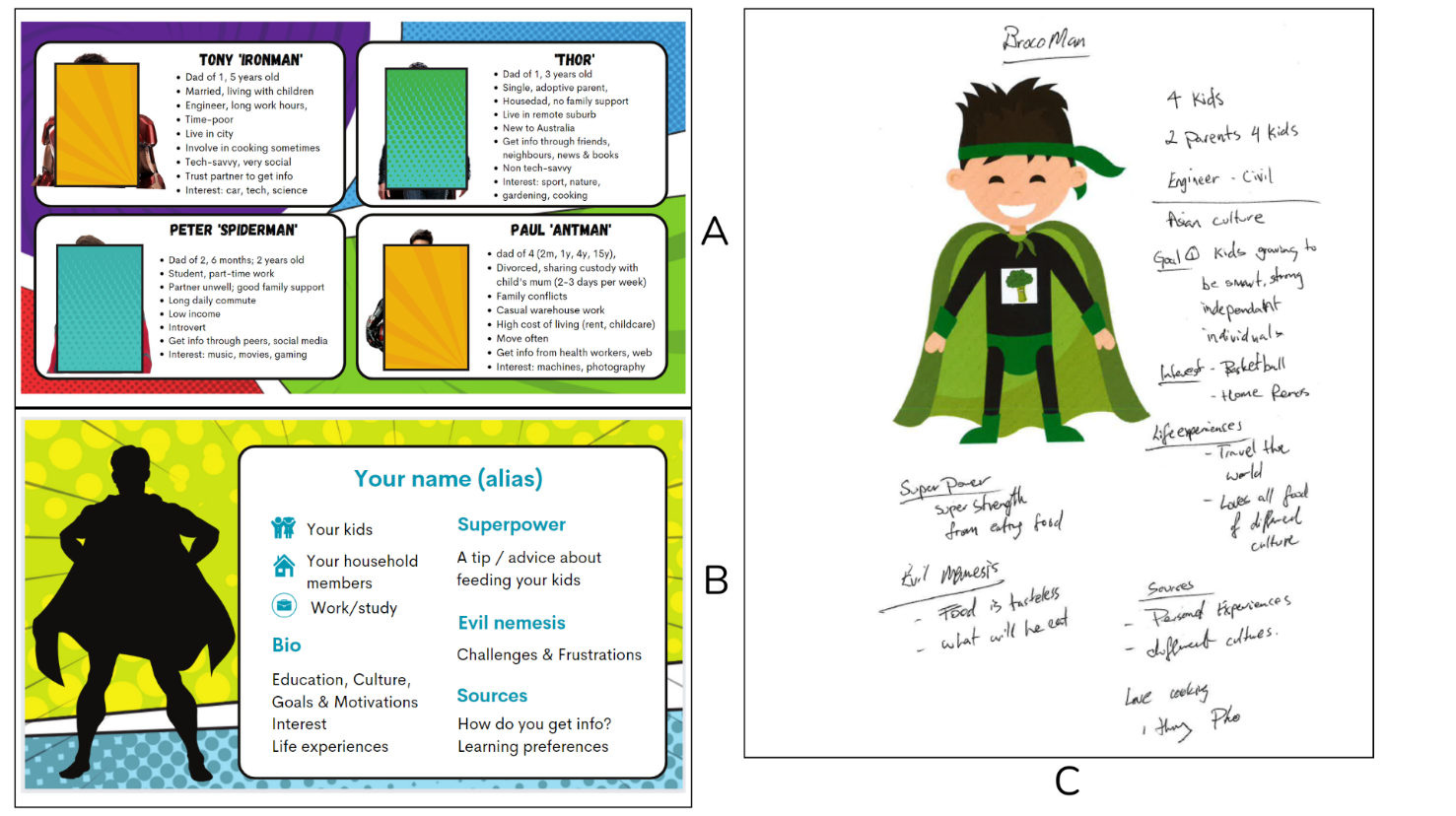


Figure 1: Exemplar slides on personas (A) exemplar personas (comic characters) incorporating findings from phases 1 and 2; (B) template slide for guiding ‘making superheroes’ activity; (C) participants’ drawing from completing ‘making superheroes’ activity (Workshop 3)

*Note:* Images were edited for publication due to copyright reason.
