# Supplementary material for: Designing Child Nutrition Interventions to Engage Fathers: Qualitative Analysis of Interviews and Co-Design Workshops
Source: JMIR Pediatr Parent. 2024 May 30;7:e57849. doi: 10.2196/57849 (PMC11176881; doi:10.2196/57849)
Supplement: Multimedia Appendix 2 [file pediatrics_v7i1e57849_app2.docx]

Multimedia Appendix 2

Design principles for child nutrition interventions with descriptions and illustrative quotes.

| Design principles | Illustrative quotes from transcripts and artifacts (i.e., chat conversation, presentation slides) |
| --- | --- |
| Father specific,  Child centered | Dad Specific - not something women… I can really agree that a lot of content is targeted towards women, it's a very stereotype 1950s housewife…So I think something community based. Facebook groups are classic example where other fathers or the person that plays that role can communicate, share ideas, and help sort of growth. (W3, Thor)  Dads will empower children by fostering a positive mealtime environment, encouraging healthy eating habits, and actively engaging in the food related aspects of family life., ensuring the kids develop a balanced and positive relationship with foods. (W1, Captain Aus) |
| Empowerment and Collaboration | Empowering - if there is something that builds confidence through simple and easy methods…Sharing space, just a collaborative space to share, so you can interact with other people…I think fathers’ groups would be best, dads empowering dads. Most females would empower too but there could be some who don't empower as much. It might feel safer for guys to be talking to guys (W1, Superdad)  It's sort of gamifying, within 6 months, 12 months you gain the competence. You then sort of become like a champion of this thing, so when you add your group of friends in this app [and] when they come to this point they need help on, hop in… they can reach out and get that work. (W2, Flash)  It's sort of sharing ideas as well as building confidence, which I think was raised earlier about learning new things and sort of having confidence in yourself to do this and that, that sort of channels is powerful in getting that…(W2, Dr. Strange)  You could have like, a family login, for someone like [participant’s name], who's divorced, that way the whole family can still be involved, like the mom can still log in from the other side and see what's been going on, the dad can log in ‘Oh, What did mom do last week?’ It could sort of act like a bit of a record as well. (W3, Thor) |
| Actionable and accessible strategies | Probably something on a web page with like ‘how to’ videos from dads and how they would do those things, the positive role modelling, active engagement, and effective communication. Like it's all good having the ideas, but then like putting it into practice, sometimes it's a bit harder for some families compared to others. (W1, Captain Aus)  What I want is to know all the ingredients I've got, so that I could shut them in an app and click create, and it will give me options. Like I don't know what basics you should have in the cupboard, right? so you can have vegetables and meats, but I don't understand how to build a base…Basic food cooking would be important, which is why I think a tech manual might help, maybe that's how guys think about things. (W2, Flash)  Making things like cookbooks and ingredient lists a bit easier for time critical families when they get home and they just need to get things done and maybe laid out in a better format. (W2, Panther) |
| Multiformat implementation | I've got the ease of access, so one forum is not going to work for everyone. It would have to be multiple forms. If you had an app, not everybody's going to use it. If you had a website, not everybody's going to use it. If you had workshop, not everybody's going to use it. have to be available on multiple platforms. (W3, Captain Wellness)  They'll have to be in-person., If you just watching it, they might inspire you, but you don't get to do it. If you're gonna do a workshop, do it in person, at least you can bring the child along as well. If you gonna have a TV show, have a TV show [with dads and children]. (W3, Brocoman)  The simple messages on the front of fridge magnets I'm pretty sure they would work. Everyone's always looking at the fridge whether we're going for a beer or getting the milk out of the fridge for the kids' breakfast. So I'd like those ideas. (W1, Captain Aus). |
| Culturally appropriate | I think the classic app, website…that you're able to input your own foods, and some sort of machine learning, AI that is using Australian ingredients and Australian seasons, could provide recommendations and build a weekly meal plan… (W2, Dr. Strange) |
| Tailored to child’s age | An app and web domain that was personalized and actionable with parenting tips and resources based on individual child development stages and needs. (W3, Captain Wellness) |
| Targeted promotion | Maybe famous chef to endorse that …because they themselves are dad and they are known for their high-quality food. Someone that is known in public for good food… [they] don’t just advocate that these are good quality recipes [but] they can help with the tips…If I were looking at like CSIRO it's a bit too ‘sciency’…I also think Facebook in this current day and age, the best place to build a community. Because most people are on Facebook, you see your friends and you want to be a part of that, it will drive you to join the community and then get into the app. It's a bit of advertising, marketing…[Also] I think places like daycares, schools kindergartens to communicate, and places like sporting events, sporting swim centres, maybe soccer, where a father or mother, parents would take their child on regular basis. (W2, Dr. Strange).  I wonder if part of it is you get it to dads through mom's sometimes. Maybe they're like ‘Hey, do you just want to help out more in the kitchen’, you sort of siloed in. Also, I'd be more inclined to trust academics and scientists. That's where I am. (W2, Flash) |
